# Development of a nomogram for predicting the intraperitoneal rupture of necrotic collection in patients with necrotizing pancreatitis

**DOI:** 10.3389/fmed.2025.1607829

**Published:** 2025-08-01

**Authors:** Gang Li, Yang Liu, Zirui Liu, Minchun Bu, Kaiming Li, Jie Yang, Jingzhu Zhang, Jing Zhou, Bo Ye, Lin Gao, Zhihui Tong, Weiqin Li

**Affiliations:** Department of Critical Care Medicine, Center of Severe Acute Pancreatitis (CSAP), Jinling Hospital, Medical School of Nanjing University, Nanjing, China

**Keywords:** necrotizing pancreatitis, intraperitoneal rupture, clinical characteristics, nomogram, predictive model

## Abstract

**Objectives:**

Intraperitoneal rupture of necrotic collection (IRNC) is an extremely rare and severe complication in patients with necrotizing pancreatitis. This study aimed to describe the clinical characteristics of intraperitoneal rupture of necrotic collection and develop a distinct model for predicting the risk of IRNC.

**Methods:**

This is a single-center, retrospective study. All consecutive patients with a primary diagnosis of necrotizing pancreatitis between January 2017 and December 2018 were screened. A nomogram was developed by multivariable logistic regression, and predictive discrimination, calibration, and clinical applicability were tested.

**Results:**

Of the 181 patients included, 20 (11%) patients developed intraperitoneal rupture of necrotic collections spontaneously during hospitalization (IRNC group). Compared with the non-IRNC group, patients in the IRNC group received more emergency open necrosectomy, and had a higher incidence of major complications and longer hospital stays. Variables associated with intraperitoneal rupture of necrotic collections included body temperature, total protein, white blood cell, and fibrinogen degradation products. A clinical prediction nomogram was developed for predicting rupture, and it exhibited better discrimination than the currently available parameters, with an area under the ROC curve of 0.897 (95%CI: 0.835–0.958).

**Conclusion:**

We have developed a nomogram to identify patients who are at risk of IRNC. Further studies are needed to externally validate and include more data to enhance the applicability and generalizability of the nomogram.

## Introduction

Necrotizing pancreatitis, defined as necrosis of the pancreatic parenchyma and/or extra-pancreatic fat tissue, occurs in approximately 20 to 30% of patients with acute pancreatitis (1). According to the revised Atlanta classification criteria, necrotic collections are classified into acute necrotic collections (ANCs) (not fully encapsulated) and walled-off necrosis (fully encapsulated) (2). International guidelines advocated postponement of interventions for necrotic collections until they have become encapsulated, such as the process that usually takes 4 weeks (3–5). However, some unexpected complications may arise during the postponement, including acute hemorrhage (6), spontaneous perforation, and/or fistulization (7). Of them, free rupture of the retroperitoneal necrotic collection into the peritoneal cavity is a life-threatening complication, and may cause pancreatic ascites, severe peritonitis, multiple organ failure, and even death.

Intraperitoneal rupture of necrotic collection is extremely rare and has been described sporadically in case reports previously (8, 9). The incidence, clinical characteristics, outcomes, and treatment strategies of this specific condition are poorly understood. In addition, these patients have a high risk of rapid clinical deterioration; hence, early triage and intensive monitoring are warranted. Even in the subgroup of symptomatic patients with ANCs, it remains unknown whether the manifestations and outcomes of patients with intraperitoneal rupture are similar to those of patients undergoing interventions due to other reasons, for instance, infection of necrotic collections.

The current study aimed to describe the clinical characteristics and outcomes of intraperitoneal rupture of necrotic collection in a cohort of patients and develop a distinct model for predicting the risk of intraperitoneal rupture in patients with necrotizing pancreatitis.

## Methods

### Patients

This study is a single-center, retrospective study conducted at the Centre of Severe Acute Pancreatitis, Jinling Hospital. Data were retrieved from an electronic database named AP-Database, which was approved by the institutional review board of Jinling Hospital (2019NZKY003-01). Each participant signed an informed consent form before the study. All consecutive patients with a primary diagnosis of necrotizing pancreatitis admitted between January 2017 and December 2018 were screened. Patients who received invasive interventions for ANCs were included. The indications for interventions of ANCs included infection, clinical condition deterioration, persistent unwellness, intraperitoneal rupture of necrotic collections, and gastric outlet obstruction (10). Key exclusion criteria were chronic pancreatitis, pregnancy, and previous intervention for necrotizing pancreatitis before admission.

Patients with intraperitoneal rupture of necrotic collections were defined as the **rupture group**, and patients without rupture were defined as the **non-rupture group**.

### Diagnosis

The diagnosis of acute pancreatitis and ANCs were made according to the revised Atlanta classification (2). The diagnosis of intraperitoneal rupture of necrotic collections was based on clinical signs, laboratory test and imaging manifestations: (i) the presence of peritonitis, including the sudden abdominal pain, the rapid appearance of ascites, peritoneal irritation signs and/or rapid clinical deterioration, and the presence of new-onset organ failure or the worsening of existing organ failure; (ii) The amylase level in ascites was remarkably elevated; (iii) The manifestations of rupture of necrotic collections into peritoneal cavity showed the contrast-enhanced CT, for instance, the decrease in necrotic collection size (11). In all included cases, the diagnosis of IRNC was strictly established prior to any intervention targeting acute necrotic collections. Furthermore, ascitic fluid amylase levels were measured via paracentesis of free peritoneal fluid, without requiring drainage of the actual acute necrotic collections.

### Data collection

The demographic characteristics, including age, gender, body mass index (BMI), and etiologies; clinical features, including disease severity, Acute Physiology and Chronic Health Evaluation II (APACHE II) score, sequential organ failure assessment (SOFA) score and computed tomography severity index (CTSI) at admission; incidence of complications, including severe bleeding, gastrointestinal fistulas, new-onset organ failure, interventions for necrotizing pancreatitis, and clinical outcomes of each patient were extracted from the database. Serum parameters, including the levels of serum creatinine, urea, total bilirubin, alanine aminotransferase (ALT), total protein, hemoglobin, platelets, white blood cells (WBCs), d-dimer, fibrinogen degradation products (FDPs), antithrombin-III (AT-III), C-reactive protein (CRP), and body temperature at admission, were also collected from medical records.

### Statistical analysis

Statistical analysis was conducted using SPSS, version 22 (IBM Corp., Armonk, New York, United States) and R Version 4.0.3 (R Foundation for Statistical Computing). The normality of continuous variables was examined by the Kolmogorov–Smirnov test. Continuous variables were expressed as median (interquartile range, IQR) and analyzed by the Mann–Whitney U-test. Categorical variables were described using frequency and percentage and analyzed by a chi-square test or Fisher’s exact test as indicated.

We first performed a screening of candidate predictor variables using univariate logistic regression analysis. The variables potentially associated with intraperitoneal rupture of necrotic collections (*p* < 0.1) were selected for our subsequent multivariate logistic regression model construction. We assessed multicollinearity among candidate predictor variables using correlation heat maps (Supplementary Figure S1) and variance inflation factor (VIF). Significant correlations or VIF values of > 10 indicated significant multicollinearity. The candidate predictor variables are included in the backward selection stepwise method multivariate logistic regression model, and the optimal predictor and final model are selected according to the Akaike information criterion (AIC). A dynamic nomogram was developed as a tool for the visualization and clinical application of the model. Predictive discrimination, calibration, and clinical applicability were tested by the area under the receiver operating characteristic curve (AUC), calibration plots, and decision curve analyses (DCAs). A higher AUC value indicated better discrimination performance. A non-significant calibration curve *p*-value indicated good calibration performance. All statistical tests were two-sided, and a *p*-value of <0.05 was considered significant.

## Results

### Participants and clinical characteristics

A total of 380 patients with necrotizing pancreatitis undergoing interventions were consecutively screened from January 2017 to December 2018. One hundred and seventy-four patients who underwent invasive interventions for necrotizing pancreatitis before the index admission were excluded. Patients with a previous history of chronic pancreatitis (4), in pregnancy status (12), or with key missing data (9) were excluded, resulting in a population of 181 patients that were included in the final analysis. Twenty patients who developed intraperitoneal rupture of necrotic collections spontaneously during hospitalization were included in the rupture group, and 161 patients without intraperitoneal rupture were defined in the non-rupture group.

**Figure 1:**
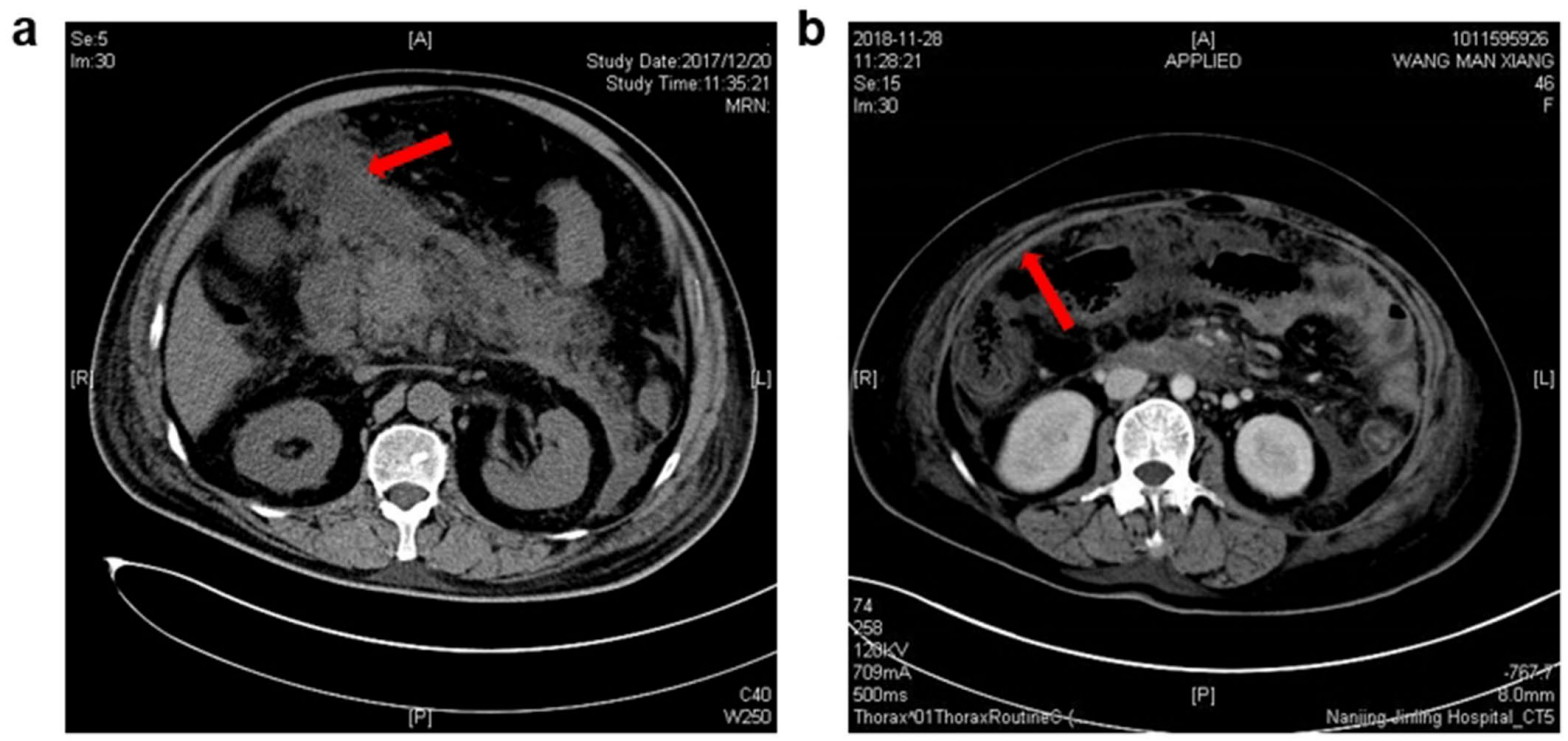
Characteristic imaging pictures of intraperitoneal rupture of necrotic collections. **(a)** From a 72-year-old man, and the CT scanning was undertaken 18 days after AP onset. Red arrow: the necrotic materials in the peritoneal cavity. **(b)** From a 48-year-old woman, and the CT scanning was undertaken 23 days after AP onset. Red arrow: Thickening of the peritoneum due to the inflammatory stimulation triggered by the rupture of necrotic collections.

Baseline characteristics of included patients with or without intraperitoneal rupture of necrotic collections are summarized in Table 1. As shown, patients in the rupture group had higher disease severities, with all the patients classified as severe AP and a higher APACHE II score at admission. Regarding the anatomical pathways of rupture, more than half of the patients had spontaneous rupture of necrotic collections through the lesser sac and then into the peritoneal cavity (55%), the second common route for rupture was the transverse mesocolon (40%), and only one patient developed intraperitoneal rupture through the splenorenal ligaments (Figure 1).

**Table 1:** Demographic data and clinical characteristics of the included patients.

Variables	Rupture group (*n* = 20)	Non-rupture group (*n* = 161)	*p* value
Gender [male (%)]	13 (65%)	108 (67.1%)	0.852
Age (years)	44.5 (19)	47 (20)	0.275
Etiology of pancreatitis			0.102
Biliary origin	13 (65%)	86 (53.4%)	
Hyperlipidemia	4 (20%)	68 (42.2%)	
Alcohol abuse	3 (15%)	2 (1.2%)	
Other causes	0 (0%)	5 (3.1%)	
BMI (kg/m^2^)	25.70 (4.03)	27.25 (4.04)	0.062
Revised Atlanta classification			<0.001
Moderate AP	0 (0%)	89 (55.3%)	
Severe AP	20 (100%)	72 (44.7%)	
APACHE II score at admission	16 (7)	13 (8)	0.013
SOFA score at admission	4.0 (3)	3 (5)	0.598
CTSI at admission	8 (2)	8 (4)	0.420
Interval from AP onset to admission	13.0 (13)	19.0 (5)	<0.001
Anatomical routes of rupture (*n*, %)			NA
Lesser sac	10 (55%)		
transverse mesocolon	9 (40%)		
splenorenal ligaments	1 (5%)		

BMI, body mass index; AP, acute pancreatitis; APACHE II, Acute Physiology and Chronic Health Evaluation II; SOFA, sequential organ failure assessment; CTSI, computed tomography severity index. Data were presented as median (IQR), IQR: interquartile range.

### Interventions and outcomes

For ANCs requiring interventions, a step-up approach was adopted (12), starting with percutaneous catheter drainage (PCD), followed by, if necessary, a videoscopic-assisted retroperitoneal debridement (VARD) and open necrosectomy served as the last resort. The mean numbers of PCD and VARD were comparable between the two groups. However, more patients in the rupture group required open necrosectomy compared to the non-rupture group (50% vs. 18.6%, *p* < 0.01).

As for clinical outcomes, no significant differences were found between the two groups in terms of mortality, while the length of hospital stay was much longer in the ruptured group than in the non-ruptured group (40.5 days vs. 31 days, *p* < 0.01), as was the total hospital cost (RMB 350.2 vs. 283.5 thousand, *p* < 0.01).

We found no significant differences between the two groups in the incidence of major complications, including bleeding, gastrointestinal fistula, new-onset renal failure, and new-onset pulmonary failure. There were higher incidences of sepsis (90% vs. 34.8%, *p* < 0.01), new-onset cardiovascular failure (85% vs. 28%, *p* < 0.01), and gastrointestinal obstruction (30% vs. 9.9%, *p* = 0.01) in the rupture group as compared to the non-rupture group. See more details in Table 2.

**Table 2:** Complications, interventions and clinical outcomes of included patients.

Parameters	Rupture group (*n* = 20)	Non-rupture group (*n* = 161)	*p* value
Incidence of complications			
Bleeding	11 (55%)	60 (37.3%)	0.126
Gastrointestinal fistula	6 (30%)	46 (28.6%)	0.894
Sepsis	18 (90%)	56 (34.8%)	<0.001
New onset RF	4 (20%)	28 (17.4%)	0.773
New onset CF	17 (85%)	45 (28.0%)	<0.001
New onset PF	6 (30%)	49 (30.4%)	0.968
Gastrointestinal obstruction	6 (30%)	16 (9.9%)	0.01
Interventions			
Interval from AP onset to first intervention	18.0 (10)	23.0 (19)	0.157
PCD	19 (95%)	160 (99.4%)	0.209
PCD times	3 (2)	3 (2)	0.594
VARD	10 (50%)	101 (62.7%)	0.270
VARD times	0 (3)	1 (3)	0.179
Open necrosectomy	10 (50%)	30 (18.6%)	0.001
Clinical outcomes			
Mortality	7 (35%)	48 (29.8%)	0.634
Length of hospital stay	40.5 (42)	31 (32)	0.007
Length of ICU stay	25 (29)	20 (21)	0.069
Total costs (RMB, thousand)	350.2 (352.3)	241.7 (283.5)	0.003

RF, renal failure; CF, cardiovascular failure; PF, pulmonary failure; AP, acute pancreatitis; PCD, percutaneous catheter drainage; VARD, videoscopic-assisted retroperitoneal debridement; ICU, intensive care unit.

### Prognostic nomogram for intraperitoneal rupture of necrotic collections

**Figure 2:**
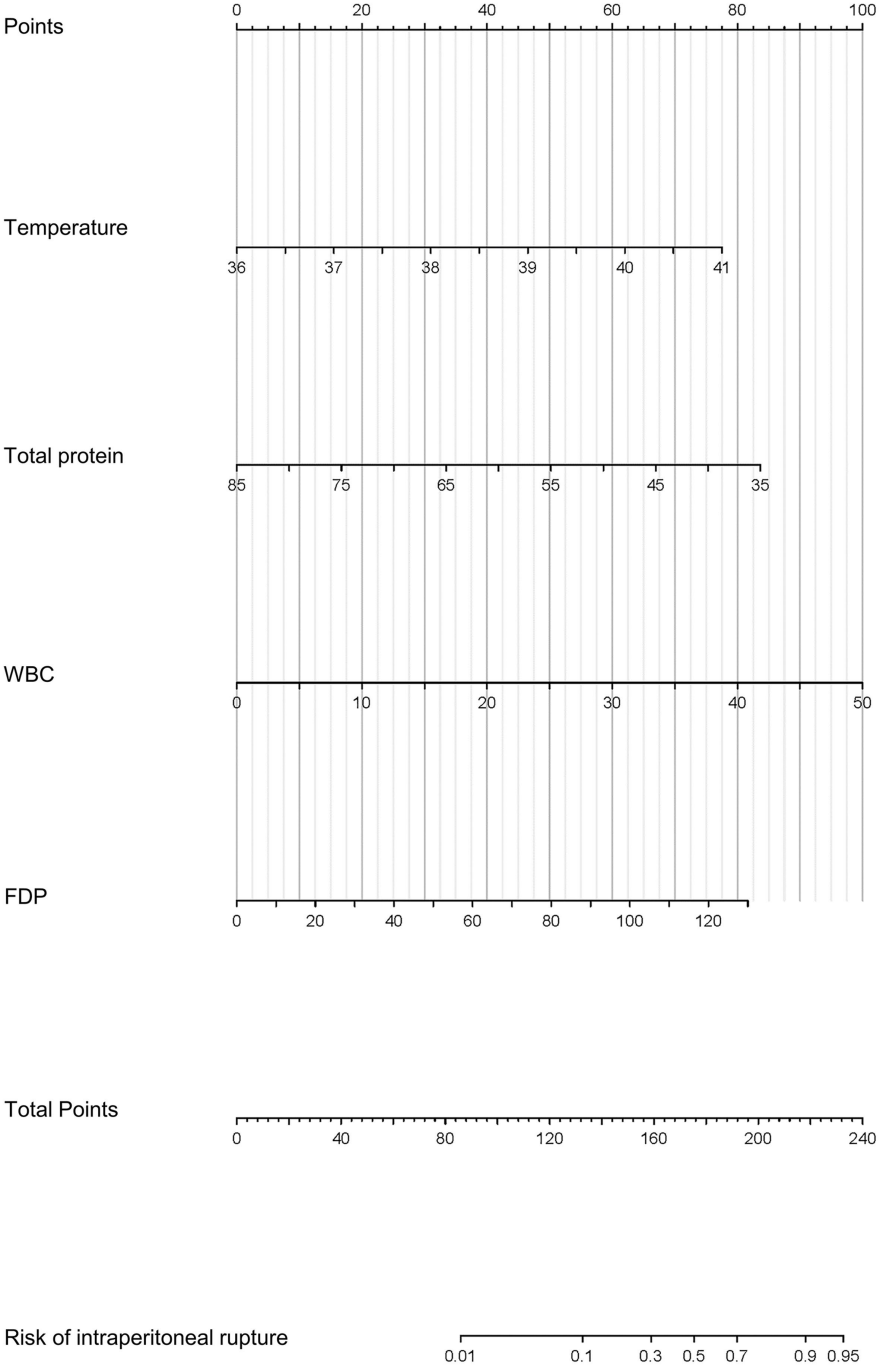
Prognostic nomogram for the intraperitoneal rupture of necrotic collections and a web-based calculator.

For nomogram development, the available clinical and laboratory data of patients were reviewed. Univariable and multivariable logistic regression showed that the optimal predictors for predicting intraperitoneal rupture of necrotic collections were body temperature, total protein, WBCs, and FDPs (Table 3). The model that incorporated the above predictors was presented as a dynamic nomogram (Figure 2) and as a web-based calculator.^1^ The nomogram was generated by assigning a weighted score to each predictor, and the individualized risk prediction probability was output by entering the value of each predictor, ranging from 0 to 1, with higher values indicating higher intraperitoneal rupture risk (13).

**Table 3:** Univariate and multivariate logistic regression analysis for incidence of intraperitoneal rupture of necrotic collections.

Variables	Univariate analysis odds ratio (95% CI)	*p* value	Multivariate analysis odds ratio (95% CI)	*p* value
Gender (male)	0.911 (0.343, 2.418)	0.852		
Age	0.980 (0.944, 1.017)	0.275		
BMI	1.125 (0.993–1.276)	0.065		
Interval from AP onset to admission	0.938 (0.899–0.979)	0.003		
APACHEII score	1.112 (1.020–1.213)	0.016		
Body temperature	3.438 (1.766–6.693)	0.001	2.217 (1.054–4.662)	0.036
Urea	1.036 (0.993–1.080)	0.098		
Creatinine	1.001 (0.999–1.004)	0.301		
Total bilirubin	1.012 (1.005–1.020)	0.001		
ALT	1.000 (0.997–1.003)	0.880		
Total protein	0.897 (0.840–0.958)	0.001	0.918 (0.847–0.994)	0.035
Hemoglobin	1.022 (1.002–1.042)	0.032		
Platelet count	0.996 (0.991–1.000)	0.049		
WBC	1.115 (1.059–1.175)	<0.001	1.108 (1.043–1.178)	0.001
D-dimer	1.099 (1.022–1.181)	0.011		
FDP	1.036 (1.014–1.058)	0.001	1.033 (1.009–1.057)	0.006
AT-III	0.974 (0.950–0.999)	0.044		
CRP	1.014 (1.007–1.021)	<0.001		

BMI, body mass index; AP, acute pancreatitis; APACHE II, Acute Physiology and Chronic Health Evaluation II; ALT, Alanine transaminase; WBC, White blood cell; FDP, fibrinogen degradation product; AT-III, Antithrombin III; CRP, C-reactive protein.

**Figure 3:**
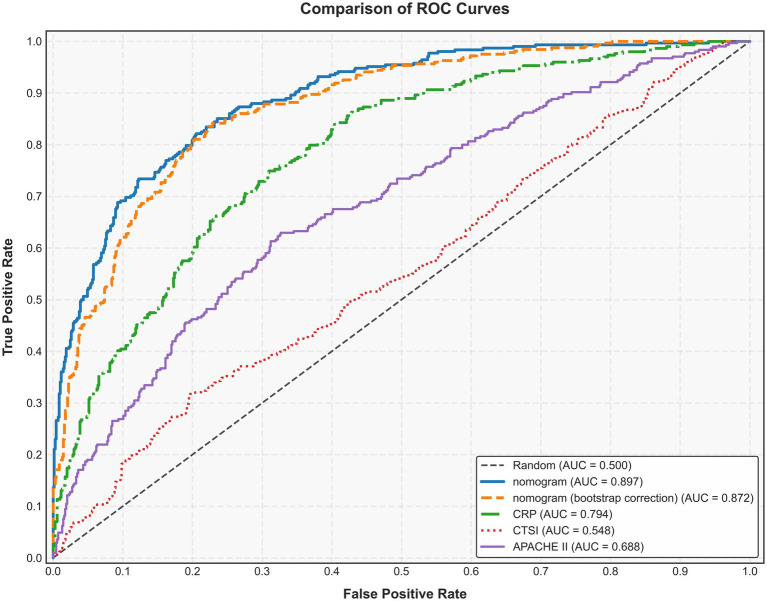
Area under the receiver operating characteristic curve (AUC) of the nomogram, APACHE II, CTSI, and CRP for predicting intraperitoneal rupture of necrotic collections. APACHE II, Acute Physiology and Chronic Health Evaluation II; CTSI, computed tomography severity index; CRP, C-reactive protein.

**Figure 4:**
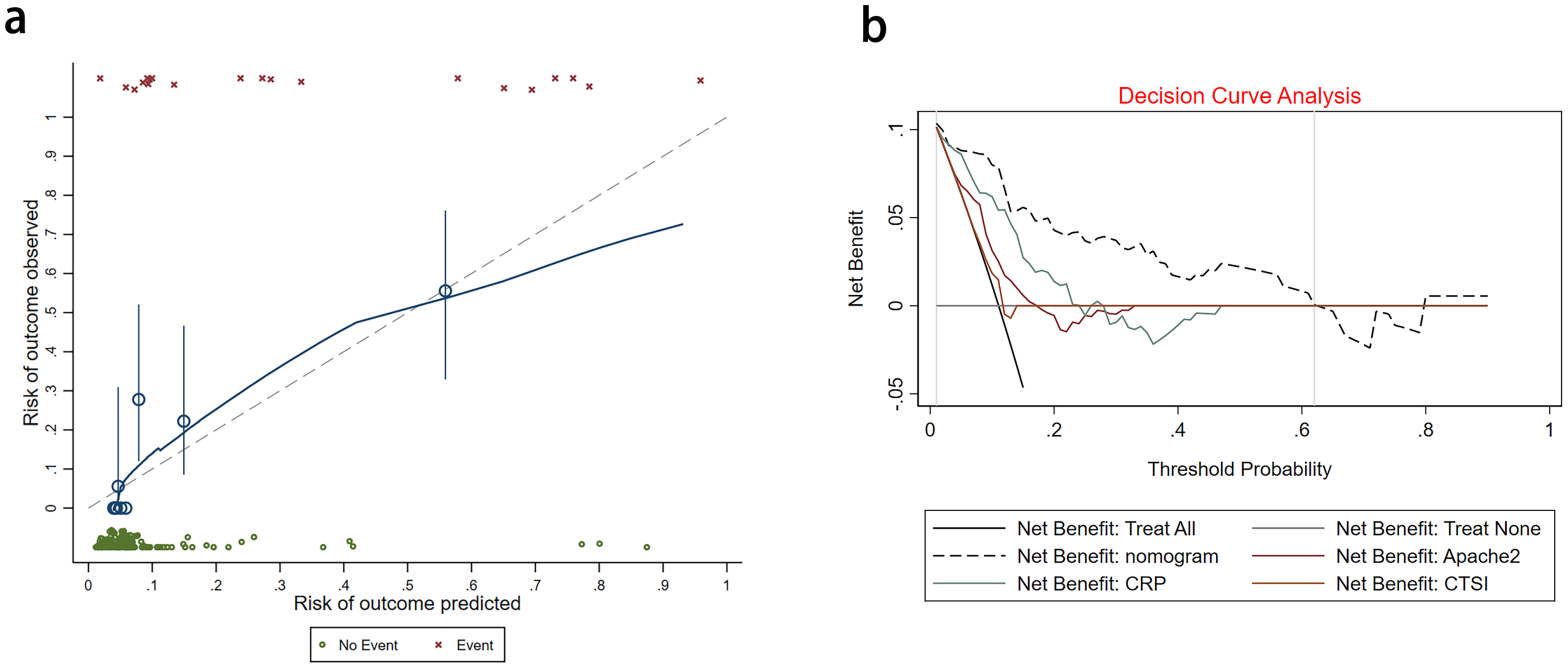
**(a)** Calibration plot for the nomogram. **(b)** Decision curve analysis for the nomogram, APACHE II, CTSI, and CRP.

We assessed the ability of our final model to discriminate patients at high risk of intraperitoneal rupture. The nomogram yielded an AUC of 0.897 (95%CI 0.835–0.958). Notably, the calibrated AUC after bootstrap correction was 0.872 (95% CI: 0.853–0.891). In comparison, the AUCs of APACHE II, CTSI, and CRP were 0.688 (95%CI 0.592–0.785), 0.548 (95%CI 0.436–0.660), and 0.794 (95%CI 0.720–0.869), respectively, indicating discrimination of the nomogram and its calibrated performance were superior to conventional parameters (Figure 3). Calibration plot revealed proper consistency between nomogram predictions and actual observations (*p* = 0.757) (Figure 4a). The DCAs revealed a net benefit superiority of the nomogram over APACHE II, CTSI, and CRP, indicating the model’s excellent clinical utility (Figure 4b). The appropriate predictive probability threshold for detecting intraperitoneal rupture using the nomogram was found to range from 0 to 62%.

## Discussion

This study demonstrates that intraperitoneal rupture of necrotic collections is an uncommon but lethal condition in patients with necrotizing pancreatitis. Compared to patients intervened for other indications, including infection, persistent unwellness, and gastric outlet obstruction, patients intervened for intraperitoneal rupture induced peritonitis or worsening of organ failure underwent more emergency open necrosectomy, and had a higher incidence of major complications and significantly longer hospital stays.

Several mechanisms may contribute to the rupture of retroperitoneal necrotic collections into the peritoneal cavity. First, activated lytic enzymes and necrotic materials might cause progressive digestion of the elastic component of the surrounding tissues (14). Second, the increase of contents in the retroperitoneal space leads to increased pressure in the retroperitoneal cavity (15). The presence of some complications, for instance, spontaneous retroperitoneal hemorrhage (16), disconnected pancreatic duct syndrome (17), infection of pancreatic necrosis would result in a dramatic increase in collection size and cause retroperitoneal localized hypertension (18). Anatomically, the lesser sac, which is composed of the hepatogastric ligament and hepatoduodenal ligament, communicates with the peritoneal cavity through the foramen of Winslow (19, 20). This is the anatomical basis for the rupture of retroperitoneal collections into the peritoneal cavity and turns out to be the most common route for intraperitoneal rupture in our study.

A clinical prediction nomogram was developed for predicting the incidence of intraperitoneal rupture in patients with necrotic collections. The nomogram comprises four variables: body temperature, total protein, WBCs, and FDPs. Elevated body temperature and WBCs are well-documented indicators of systemic inflammatory response syndrome due to bacterial translocation and cytokine release from necrotic tissues (21, 22). Hypoproteinemia correlates with disease severity in intra-abdominal infections. In IRNC, peritoneal inflammation and capillary leakage lead to protein loss into the peritoneal cavity, reducing serum total protein levels (23). Furthermore, elevated FDP levels in IRNC patients mechanistically reflect trypsin-driven hyperfibrinolysis and localized coagulopathy (24). Through trypsin-mediated Factor XII activation generating plasmin (which degrades fibrin into FDPs) and pancreatic elastase disrupting the endothelium to release tissue plasminogen activator (amplifying plasmin generation), plasmin further activates matrix metalloproteinases (degrading extracellular matrix and weakening peripancreatic tissue), while concurrent hyperfibrinolysis impairs hemostasis, promoting blood extravasation into necrotic cavities and increasing intracavitary pressure, collectively predisposing to necrosis rupture (24, 25).

The nomogram exhibited better discrimination than the currently available parameters, for instance, APACHE II and CTSI, in predicting the risk of intraperitoneal rupture. APACHE II is designed to quantify global physiological derangement (e.g., organ failure and acidosis) but lacks detailed assessment of specific local pathologies (e.g., pancreatic necrosis and pseudocyst formation) (4). CTSI primarily assesses anatomic severity (e.g., inflammation and necrosis on imaging) but may not fully capture dynamic systemic risks or evolving complications (4, 22). The prediction model also exhibited decent calibration and clinical usefulness. All indicators were available within 24 h after admission, and laboratory tests were routinely performed on patients. DCAs indicate that within the clinically useful probability threshold (Pt) range (0–62%), a lower Pt corresponds to a greater preference for conservative management, while a higher Pt corresponds to a greater preference for intervention. The optimal threshold for clinical application need not precisely align with 62%; it should be determined by balancing the consequences of missing a rupture against the risks of unnecessary intervention for unruptured cases. Hence, the nomogram may be a simple and effective tool to provide risk stratification of patients at an early stage without causing additional economic burden on patients. Further studies are warranted to validate its value in guiding clinical decision-making and optimizing the treatment of high-risk patients.

In an era where invasive intervention for necrotizing pancreatitis is increasingly postponed (26), intraperitoneal rupture of necrotic collections may be observed more often in the future because of delays in interventions. As shown in this study, the development of sepsis and subsequent new-onset organ failures were the most prominent adverse outcomes in patients with intraperitoneal rupture, which forms an urgent indication for invasive intervention. While in high-risk patients identified by the nomogram, whether an early and proactive intervention strategy, which aims to drain necrotic tissue, reduce retroperitoneal pressure, and ultimately prevent rupture into the peritoneal cavity, would benefit them remains elusive. Meanwhile, in our study, the IRNC group exhibited a significantly higher laparotomy rate (50% vs. 18.6%, *p* < 0.01) but no corresponding difference in mortality (15% vs. 12.4%, *p* = 0.73), suggesting that timely surgical intervention may counteract inherent risks. However, we acknowledge potential survival bias: Only patients who survived to receive intervention were included in the surgical cohort. Critically ill patients who died before surgery were not accounted for in surgical mortality statistics, potentially underestimating procedure-related risks.

Our study has several limitations. First, a considerable number of patients were transferred to a tertiary unit, skewing the true underlying incidence of intraperitoneal rupture. Second, the nomogram was derived from a single-institution dataset, which has an insufficient sample size. More detailed studies with larger sample sizes and external verification should be conducted to further improve and confirm the accuracy of the prediction model. Finally, although our nomogram and online calculator can identify patients at risk of intraperitoneal rupture, our study cannot determine whether an appropriate management strategy for these patients would improve outcomes.

## Conclusion

Intraperitoneal rupture of necrotic collections is an uncommon but lethal condition in patients with necrotizing pancreatitis. Compared to patients without intraperitoneal rupture, patients with intraperitoneal rupture underwent more emergency open necrosectomy, and had a higher incidence of major complications and significantly longer hospital stays. We have developed a nomogram and online calculator that can be used to identify patients who are at risk of intraperitoneal rupture of necrotic collection. These patients might benefit from early triage and close monitoring.

## Data Availability

The raw data supporting the conclusions of this article will be made available by the authors, without undue reservation.

## Footnotes

^1^ https://rubyly.shinyapps.io/DynamicNomogram/

## Supplementary material

SUPPLEMENTARY FIGURE S1 Multicollinearity was assessed among candidate predictor variables using correlation heat maps.
