# Supplementary figures and images for: Development of a nomogram for predicting the intraperitoneal rupture of necrotic collection in patients with necrotizing pancreatitis
Source: Front Med (Lausanne). 2025 Aug 1;12:1607829. doi: 10.3389/fmed.2025.1607829 (PMC12354534; doi:10.3389/fmed.2025.1607829)

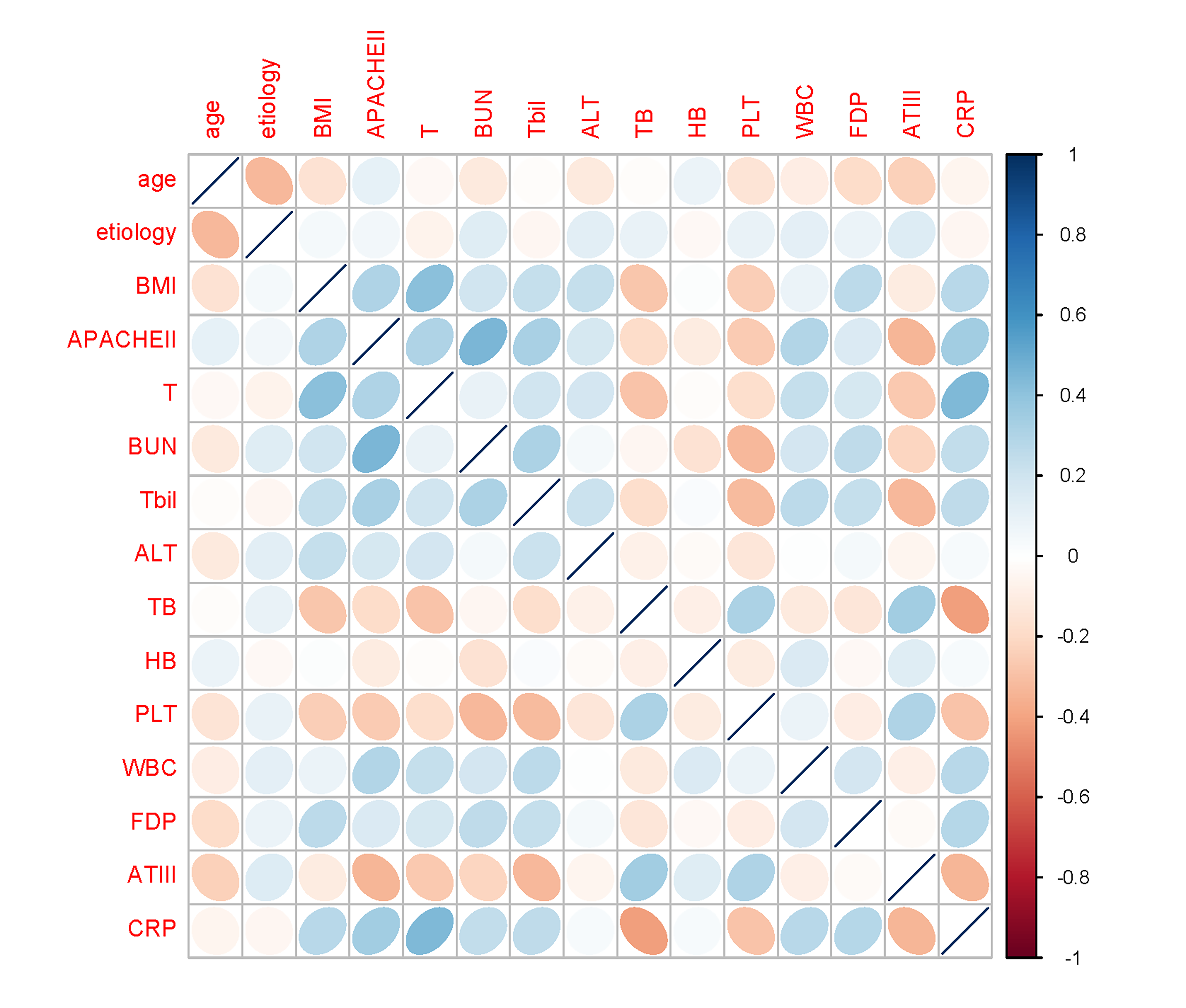

Supplement: SUPPLEMENTARY FIGURE S1 — Multicollinearity was assessed among candidate predictor variables using correlation heat maps. [file Image_1.TIFF]
